# Design and psychometrics of a tool for measuring social cognitive factors related to the preventive behaviors toward Covid-19 in the society

**DOI:** 10.1038/s41598-024-79075-x

**Published:** 2024-11-11

**Authors:** Niloofar Dadashi-Tonkaboni, Marjan Bagheri, Zeinab Gholamnia-Shirvani, Hamed Mirzaei, Mehrsadat Mahdizadeh

**Affiliations:** 1 Student Research Committee, School of Health, Mashhad University of Medical Sciences, Mashhad, Iran; 2 Department of Health Education and Health Promotion, School of Health, Mashhad University of Medical Sciences, Mashhad, Iran; 3 Department of Health Education & Health Promotion, Faculty of Medical Sciences, Tarbiat Modares University, Tehran, Iran; 4 Social Determinants of Health Research Center, Health Research Institute, Babol University of Medical Sciences, Babol, Iran; 5 Department of Education and Health Promotion, Vice-chancellery of Health, Isfahan University of Medical Sciences, Isfahan, Iran; 6 Social Determinants of Health Research Center, Mashhad University of Medical Sciences, Mashhad, Iran

**Keywords:** Behavior, Covid-19, Prevention, Psychometrics, Social cognitive theory, Health care, Public health

## Abstract

With the emergence of new strains of Covid-19, the adoption of preventive behaviors is still considered a requirement to control this disease. Therefore, considering the importance of social cognitive factors in adopting various types of preventive behaviors, the present study was conducted to design and psychometrically measure the social cognitive factors related to Covid-19 preventive behaviors. A cross-sectional study was conducted on 526 people ages 18 and over with multi-stage stratified, cluster and random sampling in Isfahan, Iran in 2022. The questionnaire was validated and formulated in three stages: designing, creating and reducing items. After collecting information and creating a bank of items, qualitative and quantitative methods were used to calculate the tool’s validity. Data analysis was done in SPSS23 software at a significance level of less than 0.05. In the present study, the average age score of the participants was 39.16 ± 11.48. The results related to the construct validity in the exploratory part show that the five factors (Outcome expectations, self-efficacy, social support, self-regulation and barrier self-efficacy) obtained have a specific value higher than one and range from 1.376 to 9.343. Correlation between factors shows that all factors have a relatively high relationship with each other (*P* < 0.05). According to the exploratory factor analysis, the final questionnaire contains 29 items and 5 constructs from socio-cognitive theory. The results showed that the thematically and technically designed tool has been prepared in an appropriate way for each of the structures and can accurately measure the structures of outcome expectations, self-efficacy, social support, self-regulation and barrier self-efficacy to explain the preventive behaviors of Covid-19 to evaluate.

## Introduction

Infectious diseases have been a constant presence throughout human history, but the emergence of new infectious diseases has accelerated in recent years, significantly affecting global health, mortality, and socioeconomic stability. Emerging infectious diseases, such as the 2019 coronavirus disease (Covid-19), have posed unprecedented challenges, leading to widespread morbidity and mortality across the globe. The World Health Organization declared Covid-19 a pandemic within four months of its initial outbreak, highlighting the virus’s rapid global spread and the urgent need for effective public health interventions^1,2^.

Iran was among the first countries to confront Covid-19, facing unique challenges in managing the outbreak. The primary strategy for controlling the disease revolves around interrupting the virus transmission chain. This requires a concerted effort to promote preventive behaviors among the public, guided by theoretical frameworks that can effectively inform health education interventions^3,4^.

To combat Covid-19, the Iranian government implemented various restrictive measures, including city quarantines and closures of public spaces. Additionally, national media and social networks played crucial roles in disseminating information about preventive measures such as wearing masks, hand hygiene, minimizing unnecessary outings, recognizing symptoms, and vaccination efforts. While government initiatives are vital in promoting public compliance with health guidelines, it is essential to recognize that individual adherence to preventive behaviors is influenced by a myriad of factors—physical, psychological, political, social, and cultural^5^. Research on past pandemics indicates that several social cognitive factors significantly affect individuals’ adoption of preventive behaviors. According to social cognitive theory, individuals are more likely to engage in new behaviors if they possess self-efficacy, behavioral capability, and positive outcome expectations^5^. The concept of “mutual determination” emphasizes the dynamic interplay between personal factors (individual traits), behavioral factors (habits), and environmental factors (social conditions), suggesting that changes in one area can influence others^4,5^.Studies have shown that fear and anxiety related to contracting Covid-19 can enhance compliance with preventive measures. For instance, research indicated that anxiety levels among Iranians regarding Covid-19 were notably higher than those reported by individuals in China, attributed to differences in social support systems^6^.Furthermore, understanding the effectiveness of preventive behaviors has been linked to adherence during previous outbreaks^3^.

Despite these insights, adherence to specific preventive behaviors during the Covid-19 pandemic in Iran has been suboptimal. For example, behaviors such as social distancing and mask-wearing were reported less frequently compared to other preventive actions. This discrepancy underscores the need for a theory-based tool aimed at evaluating health education interventions that can effectively promote adherence to recommended practices^1^.

This virus spread all over the world with high intensity, so the World Health Organization declared it a pandemic only about four months after the spread of this virus^7,8^. Iran was one of the first countries that faced this disease^9^. Since the most important and main, way to control the disease is to destroy the chain of virus transmission^10^, it is essential to pay attention to the preventive behaviours of the general public by using theories and targeted models in order to cut the chain of virus transmission^11^.

Therefore, with the spread of the Covid-19 virus and world warnings to cut off the chain of transmission, the government of Iran has applied all kinds of restrictive communication behaviours such as city quarantine and closure of public places. At the same time, through national media and social networks, it has informed people of all kinds of prevention methods, such as wearing masks, washing hands, not leaving the house except when necessary, knowing the symptoms of the disease and getting vaccinated^12,13^. Although the extensive role of the government and health policymakers in informing the public is undeniable, it should not be neglected that the adoption of preventive behaviours is influenced by other factors, such as physical, psychological, political, social and cultural factors^14^.

The studies of past pandemics indicate that several social cognitive factors have played an important role in people’s adoption of preventive behaviours^15–17^. According to the social cognitive theory, if a person has the three characteristics of self-efficacy, behavioural ability and outcome expectation, then they are more likely to decide to adopt a new behaviour and put efforts in the direction of performing that new behaviour than a person without the above characteristics^18^. The concept of “reciprocal Determination” refers to the interrelationship and mutual influence between various factors that simultaneously affect each other. In this framework, personal factors (such as individual and psychological traits), behavioural factors (such as habits and behaviors), and environmental factors (such as social and economic conditions) dynamically and mutually influence one another^19^. In other words, a change in one of these factors can lead to changes in other factors, and as a result, an individual’s behaviors and decisions are influenced by these interactions. This concept is especially used in the fields of psychology, social sciences, and public health to understand the complexities of human behavior and to prevent issues^19^.

It is worth mentioning that this theory has been successful in predicting, explaining and changing people’s behaviour in different situations^20–23^. Because from the perspective of social cognitive theory, neither internal forces nor external stimuli alone can encourage a person to act; rather, human performance is described in terms of a triple opposition in which cognitive, behavioural, and other individual factors and environmental events act together as mutual determinants of each other^18,24^.

Harper et al.‘s study showed that fear and anxiety of contracting Covid-19 will lead to compliance with preventive behaviours^25^. Additionally, in a study conducted in 2020, it was reported that the level of anxiety experienced by the Iranian people from contracting Covid-19 was at a high level, so much that it was higher than the perceived anxiety of the Chinese people. The reason for this matter was considered to be due to the existing difference in the level of social support^26^.

In 2006, based on a study conducted on the influenza epidemic as a contagious disease, the level of people’s understanding of the effectiveness of preventive behaviour in contracting the disease played an important role in adhering to the preventive behaviours^27^. Nevertheless, in a study in Iran on preventive behaviours toward Covid-19, behaviours such as social distancing, wearing a mask, and not leaving the house except when necessary were observed less than other behaviours^28^.

Therefore, according to the effective role of the studies based on social cognitive theory in explaining and promoting preventive behaviours toward Covid-19, the psychometrics of the need for a theory-based tool for the implementation and evaluation of health education interventions seems necessary. Thus, the present study was conducted with the aim to design and psychometrically measure the social cognitive factors related to the preventive behaviours toward Covid-19. It should be noted that subsequently, the psychological (self-efficacy, barrier self-efficacy and self-regulation), behavioural (Behavioural strategies), social (Social support from peers) and environmental (Perceived physical environment) structures used in the current research (in the field of studying the preventive behaviours toward Covid-19 in people) has also been implemented in the society.

## Methods

### Study design and participants

This cross-sectional study was conducted on 526 people aged 18 and over in the city of Isfahan (a historical and touristic city in the center of Iran and the third most populous city in Iran) in 2022.

### Inclusion and exclusion criteria

Inclusion criteria include: being over 18 years of age, having Iranian nationality, living in urban areas, and having normal mental and cognitive health (in particular, the absence of conditions such as depression, Alzheimer’s disease, and dementia in adults). Exclusion criteria included incomplete completion of questionnaires. It should be noted that to ensure mental and cognitive health, the participants were first asked to answer in the form of a self-report to the question “Do you suffer from cognitive and mental problems such as depression, Alzheimer’s, and dementia?“. If the answer was no, they were asked to answer questions. For people with insufficient reading and writing skills, each question was read to them by a trained person and their answers were recorded.

### Sampling and sample size

Our sampling method was designed as “stratified, cluster and random”. At first, one of the two health centers of Isfahan city (Health Center No. 1 and No. 2) was selected as a stratum. Then, 2 comprehensive urban health service centers affiliated with that center were selected as clusters. Finally, random sampling of participants was done by referring to comprehensive health service centers. This approach allowed us to ensure greater diversity and representation of the target population.

The sample size for exploratory factor validity analysis was considered to be at least ten samples for each item. A total of 526 people participated in the study based on the inclusion and exclusion criteria.

**Fig. 1:**
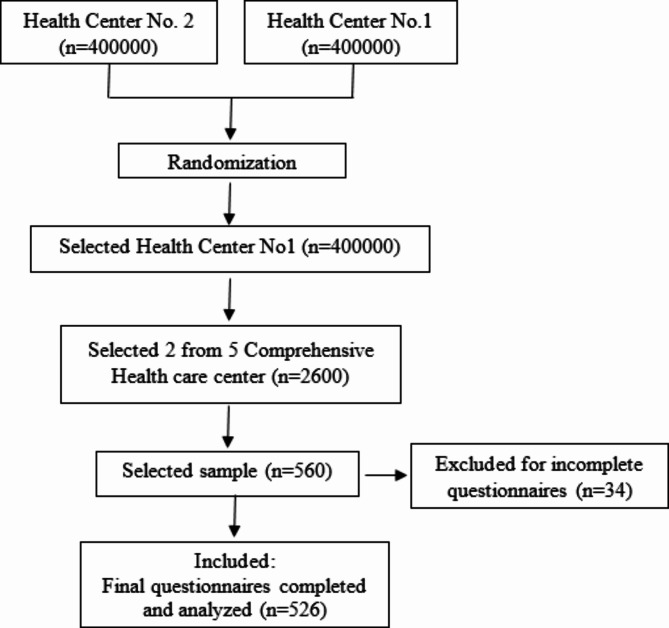
Flow diagram for sampling process.

Figure 1 shows a diagram of the sampling process, detailing the various stages of entry and exit of study participants.

### Data collection

The data were collected using a questionnaire. The questionnaire had two parts; the first part includes demographic questions such as age, gender, education, occupation, etc. The second part includes the items of social cognitive theory constructs including six items of outcome expectations (such as receiving vaccination reduces the likelihood of hospitalization and mortality due to Covid-19), six items of self-efficacy (such as: about Covid-19, I can maintain social distance to prevent the transmission of the disease to others, five social support items (such as: My family and friends encourage me to get the Covid-19 vaccine), five self-regulation items (such as: if need, I take reminder doses of the Covid-19 vaccine at appropriate intervals), and seven barrier self -efficacy items (such as: despite the need to leave the house, I adjust my daily schedule so that you leave the house less often). Responses ranged from strongly agree to strongly disagree on a Likert scale of one to five.

The questionnaire was compiled in the following process in three stages:

The data were collected using a questionnaire that was compiled in the following process in three stages:

- Step 1: Initial items were created through literature review and consultation with experts in the relevant field.
- Step 2: The number of items was reduced based on preliminary analysis and expert feedback, and it was ensured that only relevant and valid items remained.
- Step 3: Validity and reliability evaluation of the questionnaire was done.

### Validity assessment

#### Face validity

Quantitative and qualitative methods were used to assess the face validity of the questionnaire. First, in order to determine the face validity using the qualitative method, the questionnaire was given to 30 experts in the fields of research and tool making (12 experts in health education and health promotion, two experts in infectious diseases, eight nurses, three environmental health experts, Two psychiatrists and three epidemiologists. The experts were asked to comment on the cases in terms of the clarity of the statements. Also, to get the opinions of the target group, interviews were conducted with 30 of them and their opinions were applied to the questionnaire items. For quantitative formal validity, the effect score for each item was calculated as the product of the percentage of people who gave the item four and five points with the average score obtained for each item^29^. If the impact score of each item was less than 1.5, it was a candidate for elimination for the next steps.

#### Content validity assessment

In order to determine the content validity using a qualitative method, a questionnaire with 39 items was given to 30 experts to provide their correction opinions in writing and to replace the items of the questionnaire based on the criteria of using the correct grammar and appropriateness of the items and assesse the scoring of items. In addition, they could add items according to their expertise. Finally, their opinions were applied to the questionnaire. For content validity, two indexes of content validity ratio (CVR) and content validity index (CVI) 30 were used.

To determine the CVR, the panel of experts was asked to review each item based on a three-part spectrum (Necessary, useful but not necessary, and not necessary). Compared to the Lawsh Table^30^ for 30 people, the minimum and maximum CVR for questionnaire items was higher than 0.33, which led to the elimination of three items that were less than 0.33 ^31^.

To determine the CVI, the three criteria of simplicity, specificity and clarity were examined separately in a four-part Likert scale (Not relevant, somewhat relevant, relevant and completely relevant) for each one of the items by a panel of experts. The acceptance of the items was based on the CVI score of higher than 0.79 ^31^. Items in which the calculated values of CVI were higher than 0.79 were kept, seven items with CVI of less than 0.70 were eliminated, and two items with CVI of 0.70–0.79 were revised.

#### Construct validity

In this study, exploratory factor analysis (EFA) was conducted to evaluate the interrelationships between items to identify factors as constructs. It should be noted that in order to perform exploratory factorial validity analysis, a 29-item questionnaire in the constructs of social cognitive theory was given to the participants. Bartlett’s sphericity test and Kaiser-Meyer-Elkin (KMO) sample adequacy test were used. Bartlett’s test of sphericity is acceptable at (*P* < 0.05). KMO varies from 0 to 1 and more than 0.6 is acceptable. Also, criteria for factor retention were based on Eigenvalues greater than 1.

### Reliability and internal consistency assessment

To ensure the internal consistency (Reliability), the questionnaire was distributed among 30 people from the target group who were independent of the study group. In the following, Cronbach’s alpha value was calculated for outcome expectations items: 0.726, self-efficacy items: 0.903, social support items: 0.900, self-regulation items: 0.864, barrier self -efficacy items: 0.841, and the entire questionnaire had a Cronbach’s alpha of 0.955. Considering that the value of Cronbach’s alpha for all items was calculated to be greater than 0.7; as a result, they were all approved. In this research, the correlation coefficients between the scores of 60 subjects were conducted through the retest method with a time interval of four weeks between the two tests^32,33^.

The tool was completed in two stages with a time interval of one month, and then the scores obtained in these two stages were compared by using the intra-class correlation coefficient (ICC)^34^. The ICC value was calculated for outcome expectations items: 0.818, self-efficacy items: 0.936, social support items: 0.934, self-regulation items: 0.910, barrier self -efficacy items: 0.898, and the entire questionnaire had an ICC value of 0.970; in which the ICC above 0.8 showed excellent agreement^35^.

### Ethical considerations

All methods were carried out in accordance with relevant guidelines and regulations. The present study (ID: 4000725) was approved by the Ethics Committee of Mashhad University of Medical Sciences with the code of ethics of IR.MUMS.FHMPM.REC.1400.091. Other ethical matters, including explaining the research objectives at the beginning of the questionnaire, free participation in the study, assuring the participants of the confidentiality of the information and the anonymity of the questionnaires, and obtaining informed consent, were observed.

### Statistical analysis

Data analysis was completed by using descriptive statistical methods including frequency, mean and standard deviation. In the inferential part, the Pearson correlation method, t-test, ANOVA, content validity ratio (CVR), content validity index (CVI) and impact score for face validity were used. In the validity section of the constructs, EFA was conducted using Bartlett’s test of sphericity and the KMO index. Additionally, extraction methods (such as principal axis factoring) and rotation methods (such as Varimax) were employed. In the reliability and internal consistency assessment, the Cronbach’s alpha coefficient test and ICC were performed in SPSS23 software at a significance level of less than 0.05.

## Results

In the present study, the average score of the total age of the subjects was 39.16 ± 11.48. Thus, women with an average age of 36.53 ± 11.08 and men with an average age of 41.72 ± 11.29 participated in the study. In general, there were 260 women (49.4%) and 266 men (50.6%) ages 18 and over, of which 395 (75.1%) were married. In terms of the variable of education, the most frequent was the bachelor’s degree (163 people, 31%), and the least frequent was being illiterate and finishing elementary school (17 people, 3.23%). Out of 526 people who participated in the research, 208 people (39.5%) with the highest frequency were employees, and 360 people (68.4%) had an average economic status. Also, most of participants lived in the city with a frequency of 81.2% (427 people). In this study, there were 352 people (66.9%) with a history of Covid-19 infection and 486 people (92.4%) with a history of Covid-19 vaccination. Out of 526 people, only 111 people (21.1%) were healthcare workers (Table 1).

**Table 1:** Demographic characteristics of the study participants.

Variable	Subset	Frequency	Percent
Gender	Female	260	49.4
	Male	266	50.6
Job	Student	51	9.7
	Housekeeper	119	22.6
	Employee	208	39.5
	Labor	29	5.5
	Self-employment	71	13.5
	Retired	36	6.8
	Unemployed	12	2.3
Education	Literacy for reading and writing	17	3.23
	Guidance school	29	5.5
	High school	40	7.6
	Diploma and postgraduate diploma	113	21.5
	Bachelor’s degree	163	31
	Masters	137	26
	PhD and above	27	5.1
Marital status	Single	112	21.3
	Married	395	75.1
	Divorced	10	1.9
	Death of spouse	9	1.7
Economic	Weak	36	6.8
	Middle	360	68.4
	Good	126	24
	Excellent	4	0.8
History of Covid-19 infection	Yes	352	66.9
	No	174	33.1
History of the death of a family member(Covid-19)	Yes	101	19.2
	No	425	80.8
Place of residence	City	427	81.2
	The suburbs	64	12.2
	The village	35	6.7
The history of people around you who are infection with Covid-19	Yes	484	92
	No	22	4.2
	Unknown	20	3.8
History of Covid-19 vaccination	Yes	486	92.4
	No	40	7.6
Healthcare workers	Yes	111	21.1
	No	415	78.9

In this study, mutual determination with other dimensions of the questionnaire had a positive and significant correlation (*P* < 0.001), but according to the Pearson correlation test, there is no significant relationship between the age of the participants and the scores of the dimensions of the questionnaire (*P* > 0.05) and between the ages (Table 2).

**Table 2:** Correlation coefficient matrix of Outcome expectations, Self-efficacy, Self-regulation, Social Support, Barrier Self efficacy and Age Variables.

Variable	1 *r* *p*	2 *r* *p*	3 *r* *p*	4 *r* *p*	5 *r* *p*	6 *r* *p*
1-Outcome expectations	1	0.578 0.001	0.331 0.001	0.436 0.001	0.371 0.001	-0.170 0.691
2-Self-efficacy		1	0.578 0.001	0.639 0.001	0.663 0.001	-0.034 0.442
3-Social support			1	0.544 0.001	0.599 0.001	0.005 0.902
4-Self-regulation				1	0.729 0.001	-0.048 0.271
5-Barrier self -efficacy					1	0.054 0.217
6-Age						1

Significant at the level of 0.01.

Regarding the relationship between the variables, the results of the analysis of the variance test showed that there was no significant relationship between the history of Covid-19 infection, the history of the death of a family member due to Covid-19, the place of residence, the history of people around you who are infection with Covid-19 and outcome expectations, self-efficacy, social support, self-regulation (*P* > 0.05). However, the relationship between the history of Covid-19 vaccination and outcome expectations, between education and social support and between gender, education, history of Covid-19 vaccination, and self-regulation is significantly higher than others (*P* < 0.001) (Table 3).

**Table 3:** Comparison of the Mean and Standard Deviation of Outcome Expectations, Self-efficacy, Social Support, and self-regulation variables according to individual characteristics.

Variable	Subset	Outcome expectations M ± SD	Self-efficacy M ± SD	Social support M ± SD	Self-regulation M ± SD	Barrier self efficacy M ± SD
Gender	Female	24.73 ± 2.76	25.86 ± 3.28	19.33 ± 3.57	19.43 ± 3.15	24.76 ± 4.83
	Male	25.09 ± 2.85	24.24 ± 4.05	18.55 ± 3.52	22.77 ± 5.08	22.77 ± 5.08
	P-value	0.136	< 0.001	0.012	< 0.001	< 0.001
Job	Student	24.56 ± 3.04	25.50 ± 3.58	19.29 ± 3.64	19.13 ± 3.40	23.58 ± 5.20
	Housekeeper	24.51 ± 2.66	25.62 ± 3.32	19.00 ± 3.56	18.90 ± 3.29	24.62 ± 4.70
	Employee	25.59 ± 2.76	25.27 ± 3.54	18.90 ± 3.40	19.58 ± 2.81	24.20 ± 4.95
	Labor	24.93 ± 2.47	22.55 ± 5.02	17.82 ± 3.14	17.27 ± 3.62	20.75 ± 4.26
	Self-employment	25.22 ± 3.12	24.61 ± 4.68	18.91 ± 3.66	18.66 ± 3.74	24.30 ± 5.51
	Retired	24.25 ± 2.56	24.38 ± 3.71	19.01 ± 4.16	17.88 ± 3.70	22.26 ± 5.44
	Unemployed	24.58 ± 2.57	24.66 ± 4.35	19.58 ± 3.31	18.16 ± 3.71	22.41 ± 3.84
	P-value	< 0.001	0.003	0.707	< 0.001	< 0.001
Education	Literacy for reading and writing	23.00 ± 2.15	21.35 ± 5.15	16.23 ± 4.38	14.94 ± 4.30	19.52 ± 4.48
	Guidance school	23.92 ± 2.32	22.92 ± 4.06	17.92 ± 3.71	16.67 ± 3.47	21.03 ± 4.50
	High school	24.29 ± 2.28	23.53 ± 4.23	17.41 ± 3.61	17.85 ± 3.27	21.87 ± 2.75
	Diploma and postgraduate diploma	24.60 ± 2.69	25.14 ± 3.90	18.98 ± 3.63	18.46 ± 3.92	23.61 ± 4.85
	Bachelor’s degree	25.30 ± 2.59	25.22 ± 3.54	19.07 ± 3.42	19.38 ± 2.98	24.11 ± 4.79
	Masters	25.13 ± 3.14	25.97 ± 2.93	19.74 ± 3.06	19.72 ± 2.95	24.73 ± 5.08
	PhD and above	25.96 ± 3.04	25.66 ± 3.90	18.88 ± 4.31	20.66 ± 2.55	25.55 ± 5.52
	P-value	< 0.001	< 0.001	< 0.001	< 0.001	< 0.001
Marital status	Single	24.87 ± 2.81	24.88 ± 3.97	19.15 ± 3.76	18.65 ± 3.31	23.18 ± 4.94
	Married	24.96 ± 2.75	25.03 ± 3.76	18.83 ± 3.52	19.02 ± 3.28	23.84 ± 5.06
	Divorced	24.90 ± 3.92	26.44 ± 1.66	21.20 ± 3.04	20.70 ± 2.71	27.00 ± 5.45
	Death of spouse	23.11 ± 2.89	26.44 ± 3.53	18.44 ± 3.04	16.77 ± 3.59	23.44 ± 3.95
	P-value	0.272	0.529	0.175	0.050	0.129
Economic	Weak	23.69 ± 3.88	25.02 ± 4.12	19.86 ± 2.85	18.72 ± 3.70	23.80 ± 4.56
	Middle	24.94 ± 2.62	24.94 ± 3.79	18.86 ± 3.35	18.86 ± 3.28	23.49 ± 4.86
	Good	25.23 ± 2.75	25.38 ± 3.58	18.85 ± 5.12	19.21 ± 3.21	24.49 ± 5.57
	Excellent	23.00 ± 4.83	25.5 ± 5.06	20.00 ± 5.22	18.75 ± 5.12	23.75 ± 8.42
	P-value	0.015	0.706	0.393	0.744	0.302
History of Covid-19 infection	Yes	25.07 ± 2.63	24.97 ± 3.89	19.03 ± 3.53	18.91 ± 3.27	23.58 ± 4.89
	No	24.58 ± 3.07	25.20 ± 3.52	18.73 ± 3.63	18.97 ± 3.37	24.09 ± 5.37
	P-value	0.054	0.512	0.363	0.839	0.275
history of the death of a family member (Covid-19)	Yes	24.78 ± 2.99	24.85 ± 4.21	18.90 ± 3.46	18.93 ± 3.09	23.67 ± 5.04
	No	24.94 ± 2.74	25.09 ± 3.66	18.94 ± 3.59	18.93 ± 3.35	24.08 ± 5.14
	P-value	0.597	0.562	0.910	0.987	0.460
Place of residence	City	24.94 ± 2.86	24.96 ± 3.84	18.93 ± 3.51	18.78 ± 3.38	23.70 ± 5.19
	Outskirts of the city	24.71 ± 2.38	25.14 ± 3.75	18.31 ± 3.92	19.39 ± 2.96	23.57 ± 4.51
	The village	24.85 ± 2.69	25.94 ± 2.86	19.51 ± 3.46	19.88 ± 2.58	24.65 ± 4.31
	P-value	0.823	0.328	0.229	0.084	0.542
The history of people around you who are infection with Covid-19	Yes	25.40 ± 3.11	26.36 ± 3.61	20.22 ± 3.36	19.36 ± 3.71	25.86 ± 6.01
	No	24.89 ± 2.78	24.94 ± 3.79	18.86 ± 3.58	18.91 ± 3.27	23.60 ± 5.00
	Unknown	24.95 ± 2.74	26.05 ± 3.18	19.30 ± 3.34	19.05 ± 3.60	25.05 ± 4.85
	P-value	0.696	0.109	0.194	0.811	0.062
History of Covid-19 vaccination	Yes	25.07 ± 2.78	25.12 ± 3.72	19.07 ± 3.50	19.27 ± 3.03	24.06 ± 4.97
	No	22.92 ± 2.11	24.07 ± 4.26	17.25 ± 3.89	14.58 ± 3.72	20.00 ± 4.58
	P-value	< 0.001	0.090	0.002	< 0.001	< 0.001
Healthcare workers	Yes	25.19 ± 3.05	26.40 ± 3.01	19.64 ± 3.60	20.11 ± 2.73	25.74 ± 4.68
	No	24.83 ± 2.72	24.68 ± 3.87	18.74 ± 3.53	18.61 ± 3.37	23.22 ± 5.02
	P-value	0.229	< 0.001	0.018	< 0.001	< 0.001

Table 4 shows the results related to construct validity in the exploratory section, that the five obtained factors have an eigenvalue higher than one and are in a range from 1.376 to 9.343, but the age factor has an eigenvalue of less than one.

The result of rotation by Varimax method, related to Bartlett’s sphericity test, showed KMO index equal to 0.913 with 406 degrees of freedom and at *P* < 0.001 level, which indicates the adequacy of the sample size for the present study.

**Table 4:** Factors extracted from the Questionnaire along with special values.

Variable	Initial eigenvalues		
	Total	% of variance	Cumulative %
1-Outcome expectations	9.343	32.216	32.216
2-Self-efficacy	2.250	7.758	39.974
3-Social support	1.799	6.202	46.176
4-Self-regulation	1.637	5.644	51.820
5-Barrier self efficacy	1.376	4.746	60.106
6-Age	0.994	3.441	62.106

Table 5 shows the correlation between the factors, according to which all the factors have a relatively high relationship with each other (*P* < 0.05).

**Table 5:** Correlation between the factors.

Component transformation matrix					
Component	1	2	3	4	5
1-Outcome expectations	0.755	0.487	0.268	0.242	0.250
2-Self-efficacy	− 0.397	− 0.123	0.763	0.466	0.167
3-Social support	0.116	− 0.077	− 0.258	0.725	− 0.624
4-Self-regulation	0.299	− 0.729	− 0.224	0.243	0.520
5-Barrier self efficacy	− 0.413	0.459	− 0.479	0.374	0.499

Extraction Method: Principal Component Analysis.

Rotation Method: Varimax with Kaiser Normalization.

## Discussion

Controlling and preventing the spread of the Covid-19 as a pandemic infectious disease requires rapid and extensive changes in individual and social behaviors worldwide. There is a lot of evidence that shows that the adoption of preventive behaviors by most people and communities (through improving personal hygiene or social distancing measures) is generally effective in reducing the impact of the epidemic of acute respiratory diseases and reducing the speed of their transmission^36^. The present study was conducted with the aim to design and psychometrically measure the social cognitive factors related to preventive behaviors toward Covid-19 in the third most populous city of Iran, Isfahan.

In this study, there is a significant relationship between all demographic variables (except age) with barrier self-efficacy and other dimensions of the questionnaire. In line with the present study, there is a study on the topic of using an integrated social cognition model to predict preventive behaviors toward Covid-19 ^37^. However, the study of Hagger et al.^23^ howed a negative and significant correlation between preventive behavior toward Covid-19 and age. This difference is perhaps due to Hagger et al.’s special attention to adhering to social distancing in the prevention of Covid-19 infection, in which young people complied less than older adults.

In another study, the age of the participants had an inverse and significant correlation with self-efficacy, self-regulation and adherence to health practices (Diet and physical fitness) so that the average scores of the mentioned factors was higher in younger people^38^ and the reason for the difference can be attributed to the subject of the research, which was the non-communicable disease of diabetes. Also, the results of another study in Portugal showed that with increasing age, people’s participation in preventive behaviors decreases^39^.

In the present study, with mutual determination, the relationship between gender, education and history of Covid-19 vaccination was significantly higher than others. However, in another study, there was no significant relationship between any of the variables of age, gender, marital status, education level, employment status, and history of contracting Covid-19 with the adoption of preventive behaviors^40^. Perhaps the reason was the use of the health belief model.

In another study, economic status had the most significant relationship with preventive behaviors, which can be said to have two causes; (1) the exclusive attention of the researchers on two behaviors of nutrition and physical activity, (2) the non-infectiousness nature of the disease^38^ .

The correlation of the components of social cognitive theory leads to the influence of each component on the other component. As a result, it causes adherence to the desired health^41^. In the present study, in line with another study^42^, reciprocal determination (Human performance in terms of the triple interaction of cognitive, behavioral and other individual factors and environmental events) has a positive and significant correlation with other components of social cognitive theory. This finding can be placed in conjunction with the results of the study by Raude et al.^24^ in France, which showed that social cognitive factors played a more important role than sociocultural and psychosocial factors in adopting preventive behaviors.

In another study, there was a significant relationship between preventive behaviors and social cognitive theory constructs (Outcome expectations, self-efficacy and self-regulation) except for social support^38^. Additionally, in the study of Taqdisi et al., there was no significant correlation between breast cancer prevention behaviors and the structure of self-efficacy, self-regulation, and outcome expectations, and they could not predict even a small percentage of behavior changes, which was not consistent with the results of this study. Among the reasons for this issue can be the difference between the demographic characteristics of the participants of the above mentioned study in terms of education, gender, and occupation with this study, which can affect self-efficacy, self-regulation, and outcome expectations in the desired behavior^43^.

### Limitations of study

One of the limitations of this study is the reliance on self-reporting, which may introduce bias and affect data quality. Additionally, the timing of data collection was during the resurgence of Covid-19, which may have influenced participants’ responses, as public sentiment and behavior can fluctuate rapidly during a pandemic.

### Practical implications

The practical implications of this study are significant. Understanding social cognitive factors that influence preventive behaviors can inform public health strategies and interventions aimed at encouraging adherence to health guidelines during pandemics. Evaluation of interventions with valid and reliable tools can increase their effectiveness.

### Theoretical implications

Findings contribute to the theoretical framework of social cognition theory by showing the interrelationship of cognitive, behavioral and environmental factors in adherence to health behavior. It also highlights the application of behavior change theories in the development of tools for measuring health-oriented behaviors.

### Directions for future research

Future research should investigate the effect of social cognitive factors on preventive behaviors against Covid-19 and other similar diseases. Also, in future research, researchers can use this tool to evaluate the effectiveness of interventions.

## Conclusion

Based on exploratory factor analysis, the final questionnaire contains 29 items from the constructs of socio-cognitive theory. The results showed that the thematically and technically designed tool has been prepared appropriately for each of the structures and can accurately measure the structures of outcome expectations, self-efficacy, social support, self-regulation, and barrier self-efficacy in explaining the preventive behaviors of Covid-19.

## Data Availability

The datasets generated and analyzed during the current study were available from the corresponding author on reasonable request.
